# Good collaborative practice: reforming capacity building governance of international health research partnerships

**DOI:** 10.1186/s12992-017-0319-4

**Published:** 2018-01-08

**Authors:** Claire Leonie Ward, David Shaw, Dominique Sprumont, Osman Sankoh, Marcel Tanner, Bernice Elger

**Affiliations:** 1 Institute for Biomedical Ethics, University of Basel, Bernoullistrasse 28, 4056 Basel, Switzerland; 2 Institute for Biomedical Ethics, University of Basel, Basel, Switzerland; 3 Institut de Droit de la Santé, University of Neuchâtel, Neuchâtel, Switzerland; 4 INDEPTH Network, Accra, Ghana; 5 Swiss Tropical and Public Health Institute, University of Basel, Basel, Switzerland; 6 Institute for Biomedical Ethics, University of Basel, Basel, Switzerland

**Keywords:** Global Health Research, Governance, Ethics, Collaborative partnership, Capacity building, Social justice

## Abstract

In line with the policy objectives of the United Nations Sustainable Development Goals, this commentary seeks to examine the extent to which provisions of international health research guidance promote capacity building and equitable partnerships in global health research. Our evaluation finds that governance of collaborative research partnerships, and in particular capacity building, in resource-constrained settings is limited but has improved with the implementation guidance of the International Ethical Guidelines for Health-related Research Involving Humans by The Council for International Organizations of Medical Sciences (CIOMS) (2016). However, more clarity is needed in national legislation, industry and ethics guidelines, and regulatory provisions to address the structural inequities and power imbalances inherent in international health research partnerships. Most notably, ethical partnership governance is not supported by the principal industry ethics guidelines – the International Conference on Harmonization Technical Requirements for Registration of Pharmaceutical for Human Use (ICH) Good Clinical Practice (ICH-GCP). Given the strategic value of ICH-GCP guidelines in defining the role and responsibility of global health research partners, we conclude that such governance should stipulate the minimal requirements for creating an equitable environment of inclusion, mutual learning, transparency and accountability. Procedurally, this can be supported by i) shared research agenda setting with local leadership, ii) capacity assessments, and iii) construction of a memorandum of understanding (MoU). Moreover, the requirement of capacity building needs to be coordinated amongst partners to support good collaborative practice and deliver on the public health goals of the research enterprise; improving local conditions of health and reducing global health inequality. In this respect, and in order to develop consistency between sources of research governance, ICH-GCP should reference CIOMS ethical guidelines as the established standard for collaborative partnership. Moreover, greater commitment and support should be given to co-ordinate, strengthen and enforce local laws requiring equitable research partnerships and health system strengthening.

## Background

Health research is vital for better population health, equity, and national development [1]. As stated in the World Health Report 2013: ‘all nations should be producers of research as well as consumers’ [2]. However, significant constraints on skills, expertise and finance inhibit countries with limited resources from carrying out such necessary research. In response, international collaborative research partnerships have formed to bridge the health research gap in low- and middle- income countries. This has resulted in the production of new vital health data and scientific advancement, and yet persisting capacity gaps and health capabilities continue to exist between countries. Although the reasons for this reality are complex and multifaceted, one key aspect (the focus of this commentary) is achieving clarity on the role and responsibility of international health research partnerships in addressing matters of global health. The stifled progress of global health research activity is not so much a limitation in the science (although this remains a factor in respect of some diseases) but also an outcome of social and structural inequality [3]. This has affected global health priorities, including which research agendas are funded, for which target populations are health interventions designed, and how the costs, risks and benefits of research are shared. To date, partnership approaches have sustained old ghosts: north-south dependency, distorted health research priorities, weak and unprepared health care systems, underutilized local professionals and knowledge, unfair distribution of risks and benefits and insufficient access to life-saving interventions for populations most in need [4, 5]. Such factors destabilize regional development, health equity and the health of populations suffering from both endemic disease and poverty. Given this question regarding the responsibilities of ethical partnerships, this commentary explores the extent to which international health research guidelines and legislation - a crucial source of governance - require equitable partnership structures, and in particular capacity building. Fulfilling the obligation to engage in capacity building in this context means the advancement of systems, expertise and infrastructures of health research capabilities through improvement to operational, institutional and individual functions [6]. Capacity building is an ethical obligation premised on the principles of social justice and health equity; the principles respectively require the equitable distribution of risks and benefits in health research (social justice) and equal access to the resources needed to improve and maintain positive health outcomes (health equity) [7]. The objective of capacity building is to “develop individuals, organizations and societies (individually and collectively) to perform functions, effectively, efficiently and in a sustainable manner to define objectives and priorities, build sustainable institutions and bring solutions to critical national problems” [8]. As recognized by other authors, a strengthening effect through partnership may also be possible in high-income countries and across a broad variety of collaborative arrangements [9].

Where governments are unable to sufficiently establish the infrastructure, skills, and systems to conduct health research, there is an ethical duty amongst the partners of an international collaborative to build and support capacity. This is crucial in respect of addressing public health. For example, the process of building and strengthening public health capacities (in part through collaborative research) is necessary for the function and effective implementation of the International Health Regulations [10]; an international agreement of all WHO member countries designed to strengthen health security through sample collection and information sharing [11]. Failure to collaborate, or collaborate effectively, slows national and global responses to disease threats and places lives at risk, disproportionately affecting the most vulnerable [12]. The need for capacity building to establish equitable and sustainable collaborations is strongly advocated by the UN Sustainable Development Goals and the latest CIOMS ethical guidance update. Practically, the commitment of partners to capacity building is crucial for global health research to overcome inherent power differentials within collaborative research, to support local research-leadership and, to fully engage and integrate research into local healthcare settings. Fulfilling these capacity building objectives ensure that health research is able to respond to local health needs and can assure [13] the safety and health of local and global populations [12].

Increasingly, international collaborative research is being asked to consider the local interests of resource-constrained partners and the responsibility of collaborations to safeguard against the potential for structural exploitation when operating in resource-constrained settings [6, 14]. In some instances, research partnerships have actively (and explicitly) incorporated capacity building objectives in conjunction with disease and intervention research [4, 15–18]. This approach has not only been regarded as ethical but also essential for responding to local health needs, through bolstering both the health-related social structures, and addressing the urgent health needs of the affected populations [6, 19]. Research collaborations are central to the exchange of capacity. The professional and institutional links that form within multinational networks create a partnership-platform for expertise sharing, knowledge transfer and system strengthening [12]. However, attempts to establish effective capacity development approaches in global health research remain disjointed and inconsistent [20].

The commentary in this instance reflects on collaborative global health research partnerships operating in resource-constrained settings. These settings are characterised by poverty, weak healthcare systems, and high burdens of diseases; conditions that disproportionately affect disadvantaged populations and sustain vicious cycles of impoverishment [21–23]. The major concern of global health research is that despite increased investment in research programs with multiple international partners, there has been much less advancement in low- and middle-income countries accruing their own research capacity and strengthened systems of health to protect their populations [24]. This is ethically challenging and compromises the overall goals of collaborative research to improve public health and reduce global health disparities [2, 4]. For global health research partnerships to successfully respond to the health needs of vulnerable populations living in low-resource settings, an international collaborative partnership must succeed in addressing a range of complicated objectives: on the one hand generating health data and generalizable knowledge (the scientific goals), whilst on the other hand, attending to structural limitations of resources and infrastructure (the capacity goals). This requires navigating a diverse set of challenges including a range of access barriers to effective interventions and under developed health and research systems. Accounting for these different goals is important to deliver on the overall public health objective of improving conditions of health, both locally and globally.

The recognised need for global research capacity worldwide represents a new shift in thinking, with the objective being to provide all countries with health capabilities to monitor, prioritize and maintain local conditions of good health. This approach is founded on the idea that there is no global health security, without global health justice protecting health as a human right. Meeting this commitment requires locally relevant, system-integrated health research. The spread of the Ebola and Zika viruses outbreaks are just two recent cases that exemplify why local capacity is both urgent and necessary to protect the health of populations both within countries and worldwide [11, 25, 26].

## A changing governance landscape

Over time, as global health research has advanced, legislation and ethics guidelines have had to change, challenged with shifting paradigms: scientific advancement, the recognition of human rights, societal development and evolving international commitments. On the whole however international health research governance and guidance has been slow to, and inconsistent in, recognizing the principle of sustainable capacity building [27].

Endorsing the role of capacity building in partnerships has been reinforced by the United Nations Sustainable Development Goals (SDGs), 2015, (agreed upon by 193 countries) and the CIOMS guidelines (2016). These two recently published guidance documents incorporate capacity building into the standards they set, and this indicates commitment to a change in approach, at least within policy. In particular, Goal 17 of SDG states, “Enhance North-South, South-South and triangular regional and international cooperation on and access to science, technology, and innovation and enhance knowledge sharing on mutually agreed terms.” Additionally, Goal 17 also has a standalone Capacity Building Task which states, “Enhance international support for implementing effective and targeted capacity-building in developing countries to support national plans to implement all the sustainable development goals, including through North-South, South-South, and triangular cooperation.” These high-level international commitments now need to be translated into practical activities on the ground. The responsibility to do so rests with governments, legislatures, industry, NGOs and civil society.

In line with this new agenda of capacity strengthening, the latest version of the ethical research guidelines from CIOMS (2016), provides structured guidance for fulfilling capacity building objectives and equitable partnership within programmes of collaborative research [28]. Focusing in on the structure and responsibilities of partnership recognizes all stakeholders as equal, shifting the decision-making structure away from the donor-recipient dynamic often present in north-south collaborations. Addressing the ethics of the partnership in a way that goes beyond traditional research ethics (sound science, participant safety and autonomy), the CIOMS guidelines identify a crucial point: the act of entering into partnership has accompanying ethical responsibilities. This marks the need for collaborative research partnerships to contribute to sustainable capacity building activities that brings structured changes to local skills, knowledge, and systems. This is important because aiming to combat one specific health disease through collaborative research or even providing a one-off research training amongst partners will not alone secure conditions of good health for a population; co-ordinated commitment towards institutional and national capacity building is also required. Threats to health will always evolve and emerge and therefore public health requires the presence of functioning, and responsive, health and research systems. As such, CIOMS guideline states that research projects should have “local principal investigators”. This requirement ensures community consultation, context-relevant deliberative decision-making and engagement with local partners and research programs that integrate with local healthcare settings. As stipulated by the updated Guideline 8, “engaging with the community is necessary to deliver on the social value of research and respect for individual and community rights” [28]. To fulfil this objective, it is important to have partnerships that are not led solely by the funding partners, and this is achieved through ethical partnership governance – good collaborative practice - that recognises the contribution (financial or otherwise) or all partners [29, 30]. Structuring the partnership, decision-making and partner-roles through ethical governance creates an equitable collaborative environment of inclusion, transparency and accountability [29]. Capacity strengthening objectives, are not merely operational choices but ethical requirements to achieve the necessary structural changes that establish local leadership, mutual knowledge sharing, and a commitment to regional health improvements [31].

## Dominance of ICH - GCP

While the United Nations and CIOMS guidance adopts a new and more promising approach, one significant impediment acts as a barrier to translation of these essential principles into practice; the International Conference on Harmonization Technical Requirements for Registration of Pharmaceutical for Human Use (ICH) Good Clinical Practice (ICH-GCP). ICH-GCP guidelines have come to define the obligations of the research enterprise, and govern the role and responsibilities of international health research programmes. However, the adoption of ICH-GCP has been driven by the interests of the pharmaceutical industry [32]. This has perpetuated an overemphasis on clinical research guidelines that is distinct from broader objectives of collaborative research and, we argue, at odds with the objectives of the UN Sustainable Development Goals. ICH-GCP sets out to protect against human rights violations in health research by referring to the Declaration of Helsinki (DoH) and Nuremberg Code (and even in this respect its success in achieving this aim has been questioned) [33]. However, this narrow focus overlooks the partnership issues of international collaborative health research, with respect to ethical governance, social justice and promoting equity, despite these being the practical challenges of global health research today. As such, the acquired superiority, and overwhelming reliance on ICH-GCP as ethics guidance is problematic, and stands at odds with the commitment of all sectors to implement strategies that fulfil the UN Sustainable Development Goals. The aim of protecting participants from harm has become too narrowly construed to the limited framework of the researcher-participant relationship [5]. The harm or exploitation of a complex multi-stakeholder collaborative partnership can be much wider, and these concerns and risks need to be addressed with the community and the healthcare system where the research takes place [34]. At present under ICH-GCP, poorly- resourced research institutes are often instructed as service centers, rather than treated as equal collaborating partners. We argue that greater consideration needs to be given to Good Collaborative Practice, as well as Good Clinical Practice. Inequitable partnerships pose a direct threat to public health [30, 35]. Inherent injustice between partners can lead to disruption in programmes of research and public health [36, 37], compromise trust in local and global systems and [11, 12]; may even result in costly (time, money and reputation) legal consequences [38]. It is most unfortunate that the ICH did not take the opportunity of the revision of GCP in 2016 to incorporate a statement on partnership considerations and capacity building. During the review process there were repeated calls for the guidance to be updated with partnership governance and greater consideration for the needs of resource-constrained settings and yet, these safeguards have not been incorporated into the new updates [39–41].

## Recommendations: good collaborative practice

Moving forward, what changes are needed to incorporate sustainable capacity strengthening into collaborative partnerships? One is to increase awareness amongst global actors, funders, institutions, researchers and ethical review boards. In particular, it should be noted that the ICH-GCP requirements are ethically valid only to the extent that proper procedure must be followed when conducting health research; but this is only one criterion for what makes health research ethically acceptable. The ethical lens of good clinical practice needs to be widened beyond the limited protection of the participant towards a collectivist approach of good collaborative practice.

The interests of partners need not be the same, but they should be reciprocal. Therefore we recommend the process of creating a joint MoU to foster the spirit of collaboration better. We further recommend that the MoU could be a document with set criteria to direct negotiations; for example, requiring details on the constitution of the partnership and capacity strengthening in relation to the individual, institutional and operational obligations. The MOU process should be structured to facilitate communication across partners of complex collaborative research. This approach is important for defining the interests and needs of the various stakeholders; the research priorities and; the joint capacity agendas. Through co-operation partners can then form a balanced operational relationship of understanding that (fairly and transparently) allocates resources, responsibilities and project ownership amongst all partners. The partnership co-ordination set out in an MOU is crucial for realising the shared responsibilities and rewards of global health research. For example, establishing equitable partner inclusion on collaborative research protocol design, project implementation, standards of care, data handling, scientific analysis, authorship, intellectual property rights and; access to novel health research and innovation.

In the same way that an ethics committee examines an informed consent form to ensure protection of individual participants, review of the memorandum of understanding would allow oversight of capacity strengthening commitments, equitable resource allocation and evaluate the social value of the study. Procedurally, the MoU process could be stated in ICH-GCP and enforced through national legislation in much the same way as is seen with the participant informed consent process.

A second approach, beyond awareness and education, would be to co-ordinate, strengthen and enforce local laws requiring equitable research partnerships and system strengthening. Clearer agreement within guidance provisions in legislation would also have the benefit of streamlining protocol reviews across different ethical review boards and regulators, while also reducing procedural time delays, especially in multi-center studies.

The third option would be to change the ICH-GCP guidelines themselves to secure good collaborative practice through ethical partnership governance and capacity strengthening requirements. This could have been done in 2016, but the opportunity was missed. This approach honours nation state sovereignty and promotes local health research capabilities.

## Conclusion

The long-term objective for the global health community is to establish self-sufficient healthcare systems worldwide that can undertake research and respond to changing health environments. The need for autonomous and locally-led systems of health research has been better recognized with international policy campaigns, and in novel ethical research guidance. These sources have adopted various conditions requiring that local capacity building is supported in collaborative health research. Arguably, no further progress in establishing global health research systems will be achieved if the limited scope of ICH-GCP continues to take priority, as the only or overriding criteria of ethical health research. At present, this international standard of health research does not require capacity building, locally-led research or even community engagement. We argue that this needs to change. Ethical research requires that Good Clinical Practice is complemented with Good Collaborative Practice. Together, such guidance will govern international health research partnerships that nuture sustainable health research-, and health- systems in accordance with their mission; to address global health inequalities and improve local conditions of health worldwide.

## Abbreviations

CCCGH: CCGHR Principles for Global Health Research
CIOMS: Council for International Organizations of Medical Sciences (CIOMS)
COHRED: Council on Health Research for Development
DoH: Declaration of Helsinki
E6 (R1): Good Clinical Practice Guidance Document of ICH
E6 (R2): Integrated Addendum to Good Clinical Practice (GCP) of ICH
EU: European Union
FDA: Food and Drug Authority
GCP: Good Clinical Practice
GEP: Good Epidemiological Practice
GLP: Good Laboratory Practice
GMP: Good Medical Practice
ICESCR: International Covenant on Economic, Social and Cultural Rights
ICH: International Conference on Harmonization Technical Requirements Pharmaceuticals for Human Use
KFPE: Commission for Research Partnerships with Developing Countries
MOU: Memorandum of Understanding
REC SOPs: Research Ethics Committees Standard Operating Procedures
TRREE: Training and Resources in Research Ethics Evaluation
UN SDGs: United Nations Sustainable Development Goals (2015)
UNDHR: Universal Declaration of Human Rights
WHO: World Health Organization

## References

[1] Hanney SR, Gonzalez-Block MA. Organising health research systems as a key to improving health: the world health report 2013 and how to make further progress. Health Res Policy Syst. 2013;11:47. 10.1186/1478-4505-11-47 PMC3878484 24341347

[2] World Health Organization. The World Health Report 2013: Research for Universal Health Coverage. Luxembourg: World Health Organization; 2014.

[3] Denburg A, Galindo CR, Joffe S (2016). Trials infrastructure as good old-fashioned health system strengthening. Am J Bioeth.

[4] Franzen SR, Chandler C, Lang T (2017). Health research capacity development in low and middle income countries: reality or rhetoric? A systematic meta-narrative review of the qualitative literature. BMJ Open.

[5] Petryna A (2007). Clinical trials Offshored: on private sector science and public health. BioSocieties.

[6] Sewankambo N, IJsselmuiden C (2008). Responsive research in developing countries. Lancet.

[7] Gostin LO, Sridhar D (2014). Global Health and the law. New Engl J Med.

[8] UNDP. Capacity Assessment and Development in a System and Strategic Management. New York: Bureau for Development Policy, UNDP, 1998.

[9] Denburg A, Rodriguez-Galindo C, Joffe S (2016). Clinical trials infrastructure as a quality improvement intervention in low- and middle-income countries. Am J Bioeth.

[10] WHO. World Health Organization International Health Regulations (2005) France: WHO; 2005 [cited 2017 14.04.2017]. Available from: http://apps.who.int/iris/bitstream/10665/246107/1/9789241580496-eng.pdf. Accessed 4 Jan 2018.

[11] Heymann DL, Liu J, Lillywhite L (2016). Partnerships, not parachutists, for Zika research. N Engl J Med.

[12] Yozwiak NL, Happi CT, Grant DS, Schieffelin JS, Garry RF, Sabeti PC (2016). Roots, not parachutes: research collaborations combat outbreaks. Cell.

[13] Ward CL, Shaw D, Anane-Sarpong E, Sankoh O, Tanner M, Elger B. The Ethics of Health Care Delivery in a Pediatric Malaria Vaccine Trial: The Perspectives of Stakeholders From Ghana and Tanzania. J Empir Res Hum Res. 2017. p. 1–16. 10.1177/1556264617742236 29179625

[14] Angwenyi V, Asante KP, Traore A, Febir LG, Tawiah C, Kwarteng A, et al. Health Providers’ Perceptions of Clinical Trials: Lessons from Ghana, Kenya and Burkina Faso. PLoS One. 2015;10(5):1–21. 10.1371/journal.pone.0124554 PMC4416706 25933429

[15] Tupasi T, Gupta R, Danilovits M (2016). Building clinical trial capacity to develop a new treatment for multidrug-resistant tuberculosis. Bull World Health Organ.

[16] Matee MI, Manyando C, Ndumbe PM, Corrah T, Jaoko WG, Kitua AY (2009). European and developing countries clinical trials partnership (EDCTP): the path towards a true partnership. BMC Public Health.

[17] Mwangoka G, Ogutu B, Msambichaka B, Mzee T, Salim N, Kafuruki S, et al. Experience and challenges from clinical trials with malaria vaccines in Africa. Malar J. 2013;12:12–86. 10.1186/1475-2875-12-86 PMC3599886 23496910

[18] Mwangoka G, Ogutu B, Msambichaka B, Mzee T, Salim N, Kafuruki S (2013). Experience and challenges from clinical trials with malaria vaccines in Africa. Malar J.

[19] London AJ, Kimmelman J (2008). Justice in translation: from bench to bedside in the developing world. Lancet.

[20] Ottersen OP, Dasgupta J, Blouin C, Buss P, Chongsuvivatwong V, Frenk J (2014). The political origins of health inequity: prospects for change. Lancet.

[21] Reeder JC, Mpanju-Shumbusho W (2016). Building research and development on poverty-related diseases. Bull World Health Organ.

[22] Pratt B, Hyder AA (2017). Governance of global health research consortia: sharing sovereignty and resources within future health systems. Soc Sci Med.

[23] Wenner DM (2017). The social value of knowledge and the responsiveness requirement for international research. Bioethics.

[24] Ogundahunsi OA, et al. Strengthening research capacity—TDR's evolving experience in low-and middle-income countries. PLoS Negl Trop Dis. 2015;9(1):3380. 10.1371/journal.pntd.0003380 PMC4287519 25569232

[25] Shuchman M (2015). Ebola vaccine trial in west Africa faces criticism. Lancet.

[26] Schopper D, Ravinetto R, Schwartz L, Kamaara E, Sheel S, Segelid MJ, et al. Research Ethics Governance in Times of Ebola. Public Health Ethics. 2017;10(1):49–61. 10.1093/phe/phw039 PMC5444563 28567113

[27] Bernabe RDLC, van Thiel GJMW, van Delden JJM (2016). What do international ethics guidelines say in terms of the scope of medical research ethics?. BMC Medical Ethics.

[28] van Delden JJM, van der Graaf R (2017). Revised CIOMS international ethical guidelines for health-related research involving humans. JAMA.

[29] Wahlberg A, Rehmann-Sutter C, Sleeboom-Faulkner M, Lu GX, Doring O, Cong YL (2013). From global bioethics to ethical governance of biomedical research collaborations. Soc Sci Med.

[30] Chu KM, Jayaraman S, Kyamanywa P, Ntakiyiruta G (2014). Building research capacity in Africa: equity and global health collaborations. PLoS Med.

[31] Ward CL, Shaw D, Anane-Sarpong E, Sankoh O, Tanner M, Elger B. Defining Health Research for Development: The perspective of stakeholders from an international health research partnership in Ghana and Tanzania. Dev World Bioeth. 2017. p. 1–10. 10.1111/dewb.12144 28470856

[32] Lang T, Cheah PY, White NJ. Clinical research: time for sensible global guidelines. Lancet. 2011;377(9777):1553–5. 10.1016/S0140-6736(10)62052-1 21334737

[33] Shaw D, Mcmahon A (2013). Ethicovigilance in clinical trials. Bioethics.

[34] Bernabe RD, van Thiel GJ, Breekveldt NS, van Delden JJ (2016). Drug regulators and ethics: which GCP issues are also ethical issues?. Drug Discov Today.

[35] Heymann DL, Chen L, Takemi K, Fidler DP, Tappero JW, Thomas MJ (2015). Global health security: the wider lessons from the west African Ebola virus disease epidemic. Lancet.

[36] Sedyaningsih ER, Isfandari S, Soendoro T, Supari SF (2008). Towards mutual trust, transparency and equity in virus sharing mechanism: the avian influenza case of Indonesia. Ann Acad Med Singap.

[37] Fidler DP (2008). Influenza virus samples, international law, and global health diplomacy. Emerg Infect Dis.

[38] Nordling L (2015). Africa’s fight for equality - after years of second-class status in research partnerships. Afr Sci Calling Change Nat.

[39] Lang T. Fundamental problems with ICH-GCP: #4 “a camel is a racehorse designed by a committee”. More Trials. 2016. p. 1.

[40] Ravinetto R, Tinto H, Diro E, Okebe J, Mahendradhata Y, Rijal S (2016). It is time to revise the international good clinical practices guidelines: recommendations from non-commercial north–south collaborative trials. BMJ Global Health.

[41] Vischer N, Pfeiffer C, Joller A, Klingmann I, Ka A, Kpormegbe SK (2016). The good clinical practice guideline and its interpretation - perceptions of clinical trial teams in sub-Saharan Africa. Tropical Med Int Health.

